# A BAC-Based Transgenic Mouse Specifically Expresses an Inducible Cre in the Urothelium

**DOI:** 10.1371/journal.pone.0035243

**Published:** 2012-04-09

**Authors:** Tian Huai Shen, Nataliya Gladoun, Mireia Castillo-Martin, Dennis Bonal, Josep Domingo-Domenech, Daniel Charytonowicz, Carlos Cordon-Cardo

**Affiliations:** 1 Herbert Irving Comprehensive Cancer Center, Columbia University Medical Center, New York, New York, United States of America; 2 Department of Pathology, Columbia University Medical Center, New York, New York, United States of America; 3 Department of Urology, Columbia University Medical Center, New York, New York, United States of America; Kanazawa University, Japan

## Abstract

*Cre-loxp* mediated conditional knockout strategy has played critical roles for revealing functions of many genes essential for development, as well as the causal relationships between gene mutations and diseases in the postnatal adult mice. One key factor of this strategy is the availability of mice with tissue- or cell type-specific Cre expression. However, the success of the traditional molecular cloning approach to generate mice with tissue specific Cre expression often depends on luck. Here we provide a better alternative by using bacterial artificial chromosome (BAC)-based recombineering to insert *iCreERT2* cDNA at the *ATG* start of the *Upk2* gene. The BAC-based transgenic mice express the inducible Cre specifically in the urothelium as demonstrated by mRNA expression and staining for LacZ expression after crossing with a Rosa26 reporter mouse. Taking into consideration the size of the gene of interest and neighboring genes included in a BAC, this method should be widely applicable for generation of mice with tissue specific gene expression or deletions in a more specific manner than previously reported.

## Introduction

Bladder cancer is one of the major causes of malignancy-related morbidity and mortality worldwide, representing the 7^th^ most common type of cancer world wide and the 4^th^ in developed countries [1]. More than 90% of bladder cancers correspond to urothelial carcinomas, since gene mutations are believed to occur in the urothelium, thus initiating malignant transformation. Profiling of bladder cancer samples from patients has revealed frequent inactivation mutations of key tumor suppressor genes such as *PTEN*, *TP53*, and *RB* [2]. However, the causal relationship between these mutations and bladder cancer development has not been well studied in genetic mouse models of bladder cancer due to the lack of suitable transgenic mice that can be used to inactivate these tumor suppressors in the adult, since Pten and Rb are required for embryonic development, and Pten/Rb deficient mice are not born [3], [4].

The *Upk2* promoter has been used successfully in driving expression of Cre [5] and a modified reverse tetracycline trans-activator[6] in urothelium specific fashion, as well as in the generation of two transgenic mouse models of invasive and superficial bladder cancers [7], [8]. This breakthrough in bladder cancer modeling was made possible only after large-scale protein purification from bovine bladder followed by cloning of the urothelial specific *Uroplakins* [9], [10]. On view of these published works, we therefore generated a mouse line that expresses an inducible CreERT2 recombinase under 3.6 kb of the *Upk2* promoter sequence. Surprisingly, we found that CreERT2 expression was not exclusively observed in the urothelium of bladder and ureters, but also in testis and brain (unpublished observation), which is in concordance with previous reports that described the use of the same *Upk2* promoter for the generation of transgenic mice [10], [11]. Recently, it was found that there is sporadic ectopic expression of Cre in tumors developed in the skin and the lung using the same *Upk2* promoter driven Cre mouse, indicating limitations of tissue specificity conferred by the short promoter although the unexpected *Upk2* promoter activity was beneficial to the particular study [12].

To overcome this non-specificity, we reasoned that by using a much large construct such as a bacterial artificial chromosome (BAC) that contains most, if not all, transcriptional determinants of a gene, the tissue specific expression would be guaranteed. It is noteworthy that BAC-based transgenic approach has been used extensively in the field of neuroscience where complex composition of cell types in the brain demands absolutely specific transgene expression [13], [14]. Likewise, increasing number of researchers in other fields has adopted this approach to achieve cell specific gene expression [15], [16], [17], [18]. As a further demonstration of the potential of BAC-based approach, by replacing a large segment in the mouse genome with a syntenic human BAC DNA, humanized mice can be made to better modeling human diseases [19]. Indeed, using recombineering [20], we were able to precisely modify a BAC clone that contains the *Upk2* gene to generate a BAC-based transgenic mouse line with restricted expression of CreERT2 in the urothelium, which we named as *TgUICBAC*.

## Results and Discussion

**Figure 1:**
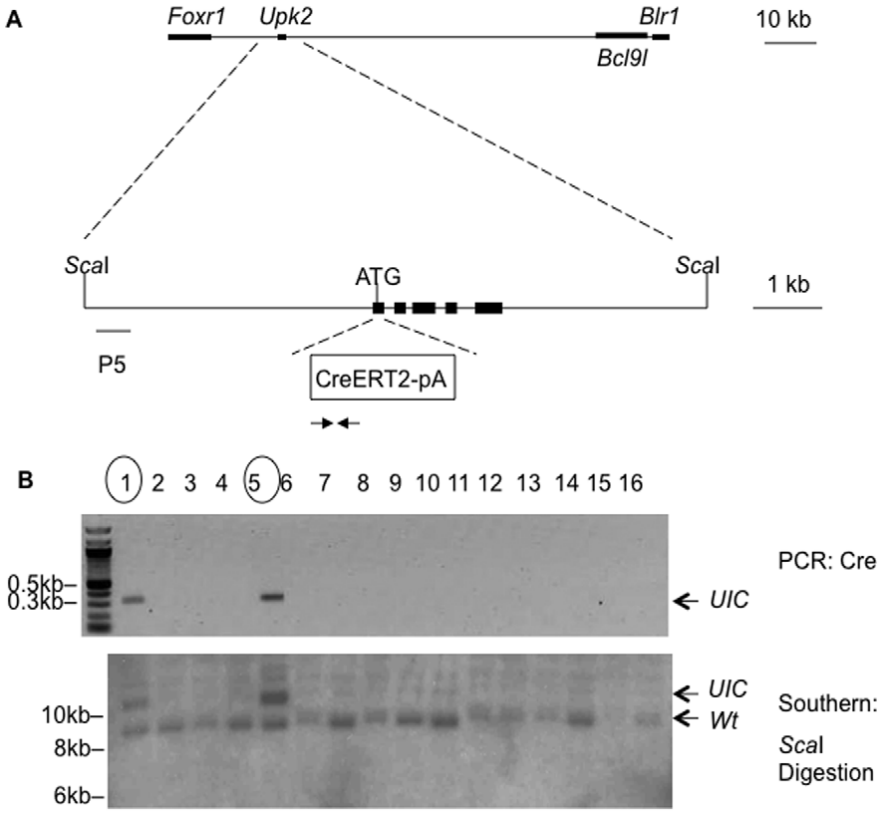
Generation of mice carrying insertion of *iCreERT2* into the *ATG* of *Upk2* gene. **A)** The BAC clone bMQ-343M5 contains the *Upk2* gene flanked by parts of *Foxr1* and *Blr1* genes, which together with *Upk2* gene are all transcribed from reverse strand, and *Bcl9l*, which is from forward strand. The size of the BAC clone insert is 82951 bp (from 44267673 to 44184722 in mouse chromosome 9). *iCreERT2-pA* is inserted into the *ATG* start of *Upk2* gene. ScaI sites and probes for southern blot (P5) are shown. Arrows indicate primers used for PCR genotyping. **B)** Mouse genotyping by PCR (upper panel) and Southern blot (lower panel). Positive founder lines (#1 and #5) are marked with circles. *UIC* stands for the *TgUICBAC* transgene.

*Upk2* is a single gene in the mouse genome, and is flanked by *Foxr1* and *BCl9l* on mouse chromosome 9. Using recombineering, we inserted a codon-improved *iCreERT2* [21] together with a polyA tail into the ATG of Upk2 translational start site of a BAC clone that contains the *Upk2* gene (Figure 1**A**). This modified BAC clone was subsequently injected into fertilized eggs of B6CBA/F1 mice to generate transgenic *TgUICBAC* mice. Two mice were identified to contain the transgene based on genotyping by polymerase chain reaction (PCR) and Southern blot analyses: Mouse #1 and #5 had 2 and 4 copies of the transgene, respectively, based on the intensity of signals on the Southern blot in comparison with that of the endogenous *Upk2* allele (Figure 1**B**). Only mouse #1 which showed a low copy number of the transgene was able to produce offspring; therefore, it was used for further characterization of the *TgUICBAC* mice. The transgenic mice showed no phenotypic alterations and bred normally, producing offspring with regular number of litters.

**Figure 2:**
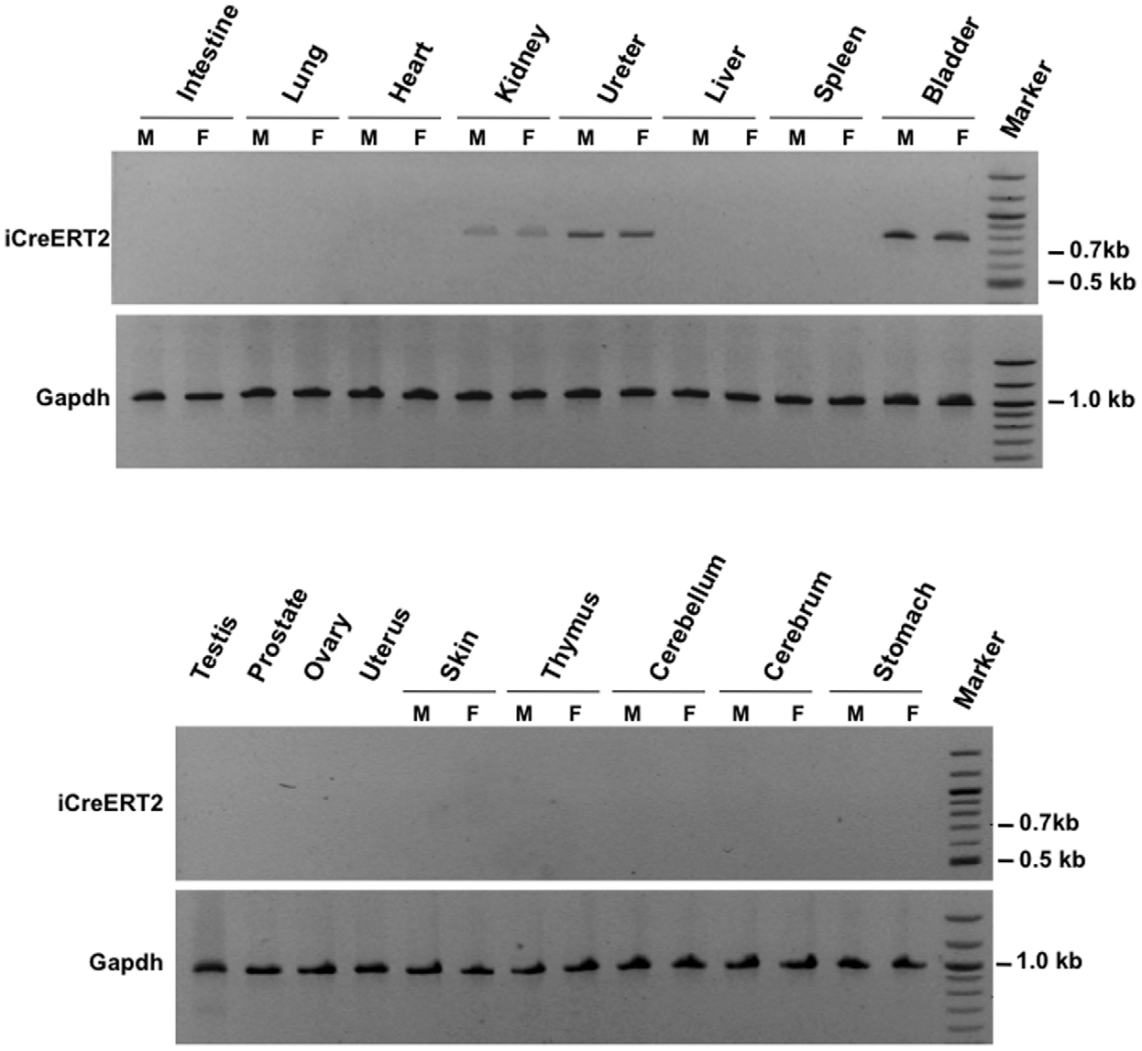
iCreERT2 expression in *TgUICBAC* mice. Total RNA from various organs of male (M) and female (F) mice were extracted, and subjected to RT-PCR analysis of iCreERT2 expression. Note that iCreERT2 expression is observed only in kidney (renal pelvis), ureter and bladder, revealing that it is specific of organs coated with urothelium. Mouse Gapdh served as a control.

**Figure 3:**
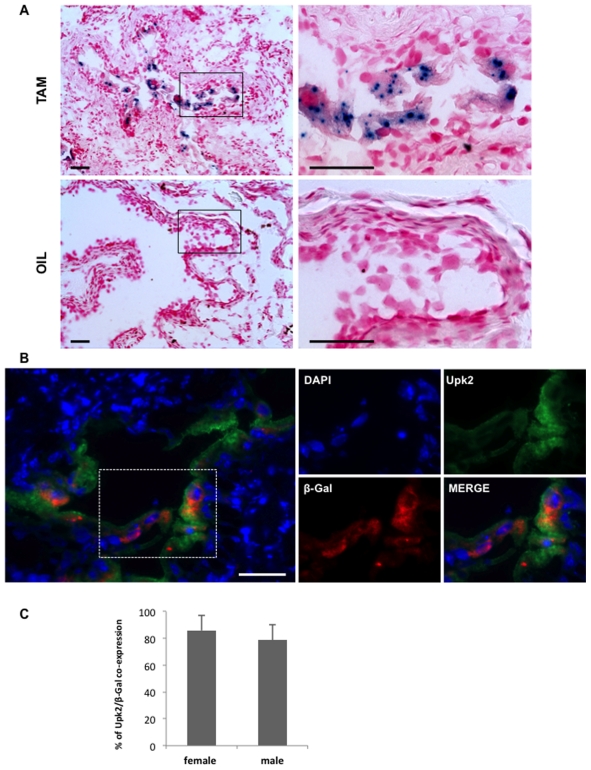
iCreERT2 activity in *TgUICBAC* mice in vivo. **A**) Eight week old *TgUICBAC;Rosa26* mice were injected with Tamoxifen for 3 days (TAM) or equal volume of sunflower oil (OIL, control group). Mice were sacrificed one week after injection, bladder sections were obtained and X-gal staining was performed. Note that X-gal blue staining is observed in the epithelial cells of the bladder in mice injected with TAM, whereas no staining is observed in the bladder of control mice. Bars correspond to 100 µm. **B)** Representative immunofluorescence analysis of Upk2 (green) and ß-galactosidase (red) co-expression in bladder urothelium of *TgUICBAC;Rosa26* mice injected with Tamoxifen. Note that most of the Upk2-positive urothelial cells show a doted staining for ß-galactosidase. Bars correspond to 100 µm. **C)** Quantification of percentage of recombination in the analyzed bladder sections from 2 female and 2 male transgenic mice illustrated by mean ± SD.

**Figure 4:**
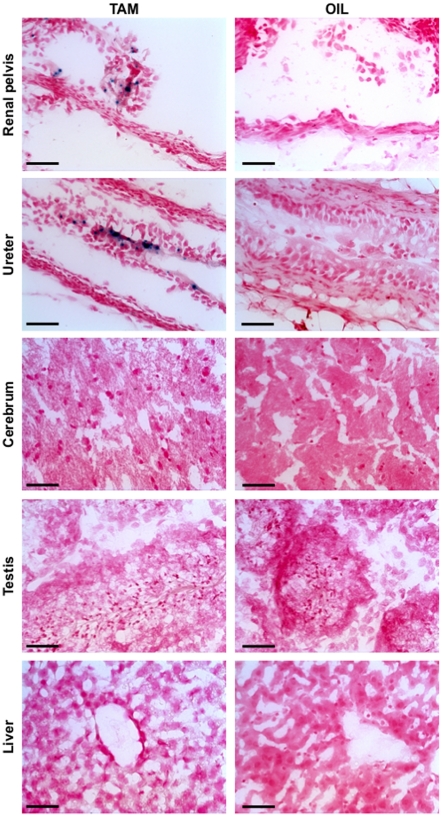
Tissue-specific activity of iCReERT2 in vivo. Eight week old *TgUICBAC;Rosa26* mice were treated as in Figure 3, sections of other organs were obtained and X-gal staining was performed. Note that X-gal blue staining is observed only in the epithelial cells of the renal pelvis, ureter (indicated with arrows) in mice injected with TAM, whereas no staining is observed in control mice or other organs of TAM-injected mice. Bars correspond to 100 µm.

To confirm that iCreERT2 expression was specific to the urothelium, total RNA was extracted from the major organs of male and female transgenic mice and subjected to RT-PCR analysis using specific primers to iCreERT2. Indeed, differently to previously reported *Upk2*-based transgenic mice [10], [11] and our *TgUPCreERT2* mice (unpublished observation), only organs that contain urothelium express the transgene, such as bladder, ureter, and kidney (renal pelvis), whereas no other organs such as brain and testis were found to express the transgene (Figure 2). To further prove the specificity and in vivo activity of iCreERT2, *TgUICBAC* mice were crossed with Rosa26 reporter mice in which a floxed stop cassette is placed before the *LacZ* gene so that LacZ expression is allowed only after the stop cassette removal mediated by Cre activity [22]. When the compound mice were administered Tamoxifen by injection, X-gal staining revealed a strong and distinct blue staining in the epithelial cells of the urothelium of bladder, whereas no blue staining was observed in bladder tissue of control mice which received only oil injection (Figure 3A). To assess the percentage of recombination, we performed double immunofluorescence using antibodies against Upk2 and β-galactosidase (Figure 3B), and quantified co-expression of these two markers (Figure 3C). This analysis revealed that recombination rate was observed in most of the Upk2 expressing cells (82.3%± 11.2%) in the four representative mice analyzed, underlining the potential use of our model. No significant differences were observed between female and male bladder recombination rates, which corresponded to 85.8%± 10.9% and 78.7%± 11.3%, respectively (p = 0.297). As further evidence of the Cre specificity, X-gal staining was also found in the urothelium of ureter, and renal pelvis, but not in other organs that do not contain urothelium in the compound mice (Figure 4). These data indicate that we have generated an urothelium-specific and tamoxifen-inducible Cre mouse line with a high recombination rate in the urothelium of both male and female.

Because the BAC clone that was used for this study included genes flanking *Upk2*, namely *Bcl9l*, *Blr1 (or Cxcr5)* and *Foxr1,* and the transgenic founder with higher copy number of the BAC was unable to breed (Figure 1), overexpression of these genes may have phenotypic consequences. Therefore, we performed quantitative RT-PCR to determine the transcript levels of such genes in the bladder of *TgUICBAC* mice that breed normally with low copy number of the BAC. As expected, the transcript levels of these three genes are significantly up-regulated in the bladder of the transgenic mice compared to those in wild type mice (Figure S1). Although there are no phenotypic changes in the established *TgUICBAC* mouse line, it is necessary to identify and separate potential effects of overexpression of the three genes on the phenotypes of interest by including proper control groups, as it is usually the case in studies of mouse modeling of human diseases.

The specificity of the *TgUICBAC* transgene expression confirms that Upk2 is an urothelium marker [23], suggesting that the short *Upk2* promoter may lack some transcriptional determinants to allow transgene expression non-specifically in other organs such as brain and testis. This mouse line that we have generated will be a critical tool for conditional inactivation of tumor suppressors specifically in the urothelium for bladder cancer modeling.

Recombineering is a homologous recombination mediated genetic engineering in bacteria [20]. It does not rely on the presence (or absence) of restriction sites that traditional molecular cloning requires. It has the capability to modify at nucleotide level precision any possible genetic modifications on a large DNA molecule, such as a BAC clone. Because of its size at the average of over 100 kb [24], a BAC clone usually covers the entire genomic structures of most genes in the mouse genome, it is perhaps not surprising that a BAC-based transgenic mouse approach as described here will provide faithful gene expression and will be the future trend in the generation of transgenic mice for various purposes.

The expression of unintended genes close to the gene of interest in the BAC as identified in this work represents a common but often neglected issue in the BAC-based transgenic organisms, which is usually the case when the size of the gene of interest is small. Neighboring genes in a BAC can be removed by recombineering provided that these genetic elements do not contribute to the specificity of expression of the gene of interest. On the other hand, if a gene is too big (such as over 200 kb), the BAC-based transgenic approach is not reliable because of the size limitations of average BACs. If these are major concerns in both scenarios, a targeted knock-in will be the best choice. In spite of these shortcomings, the BAC-based transgenic approach is faster and easier than gene targeting, and even advantageous when adding an extra-targeted allele is less desirable than a simple transgene in the complicated breeding scheme to get multiple targeted genes in a single mouse.

## Methods

### Ethic Statement

All procedures involving mice were conducted in accordance with National Institutes of Health regulations concerning the use and care of experimental animals, and according to the approved animal use and care protocol (AAAA7254) at the Herbert Irving Comprehensive Cancer Center of Columbia University.

### Mouse Strains

Rosa26 reporter mice [22] and C57BL/6 mice were purchased from Jackson Laboratory. The *TgUICBAC* founder mice were in a B6CBA/F1 background, and founder line #1 was backcrossed with C57BL/6 for more than 10 generations. The established *TgUICBAC* mice have normal appearance and behavior.

### BAC Transgene Construction

A mouse BAC clone with the *Upk2* gene in the middle and flanked by large genomic regions at either side was obtained from an annotated 129Sv BAC library [24]. The BAC DNA was purified using a regular plasmid DNA mini-prep kit (Qiagen, Valencia, CA) and was used as a template to verify the content by PCR using primers that amplify a segment of *Upk2* gene. The PCR product was sequenced and confirmed. The *iCreERT2* cDNA and a polyA tail together with a flrted selection marker *Amp* coding for resistance to Ampicillin was amplified using high fidelity DNA polymerase (New England Biolabs, Ipswich, MA). Primers were 100 nt long and allowed the amplification of the *iCreERT2* cassette flanked by sequences at both sides of the *ATG* start site of the *Upk2* gene. The primers were synthesized by Invitrogen (Invitrogen, Carlsbad, CA). The PCR product was purified and completely sequenced to verify that there were no un-intended mutations. Then 100 ng of the purified PCR product and 10 ng of the BAC DNA were co-electroporated into 105 competent cells that are capable of recombineering [25]. After Chloramphenicol and Ampicillin selection, the survived clones were picked and verified by PCR and sequencing of the PCR product to verify the location of the insertion. Restriction digestion with SpeI was performed to confirm that there was no re-arrangement of the modified BAC clone compared to its parental clone. Then the *Amp* selection cassette was removed in Flpe expressing bacteria. Finally, the *loxp* site present in the BAC clone was deleted by recombineering using *Amp* as a selection marker. The final BAC DNA was purified using a large construct purification kit (Clontech, Mountain View, CA), and subject to microinjection into fertilized eggs of B6CBA/F1 mice at the transgenic core facility of the Herbert Irving Comprehensive Cancer Center of Columbia University. Primers used in these procedures are summarized in Table 1.

**Table 1:** Primer sequences for generation and characterization of *TgUICBAC* mice.

Primers (5′→3′) and Applications
ATTGGCCCCAGGAAACCCCAGCCTGTCAGCACCTGTTCCAGGATCCAGTTCCCAGCGCAGTATGgatatctccaacctgctg (forward)GAGCCAGGACAGCCAGCAGAATCAGGATCAGGGGCAAGGTCTGGACAGGCAGTGTGGATGCgctagccgttaattaagaag (reverse)Using *pIndu-perfect* as a template to get a 3498 bp *iCreERT2* Cassette. Capital letters are from *Upk2* sequence, lower-case letters are from *iCreERT2* sequence.
CTGAGGCTACAGTGCCCAAG (forward)GTCTAGCGCTCTGAAACCCTC (reverse)Using modified BAC DNA as a template to get a 3772 bp (or 274 bp for unmodified BAC) product.
cttatcgatgataagctgtcaaacatgagaattgatccggaacccttaatTCTTAGACGTCAGGTGGCAC (forward)ccgatgcaagtgtgtcgctgtcgacggtgaccctatagtcgagggacctaTCACGTTAAGGGATTTTGGT (reverse)Using *pTAMP* as a template to get a 860 bp *Amp* cassette to delete the loxp site in BACe3.6. Lower-case letters are from BAC3.6 sequence, capital letters are from *Amp* sequence.
ATGGATATCTCCAACCTGCTG (forward)AGATCTCCTGTGCAGCATG (reverse)Using tail genomic DNA to get a 312 bp product for *TgUICBAC* transgenic mice PCR genotyping.
TGCTCCCAGGTGGTGGATCTC (forward)CTCAAAGCGGACCTCCTGTTC (reverse)Using BAC DNA as a template to get a 389 bp probe for Southern blot genotyping. Using the probe and *Sca*I digestion to get 8869 bp and 11196 bp for transgenic and Wt allele on Sothern blot, respectively.
CGCGAGAGGAGTGTGTCTG (forward)CTCTGAATCGAGGGATGGAGT (reverse)qRT-PCR for *Bcl9l*
AGGCCGAGCAGAAACTTGC (forward)GGCTTAATGTCTGGACCATCTTT (reverse)qRT-PCR for *Foxr1*
TGGCCTTCTACAGTAACAGCA (forward)GCATGAATACCGCCTTAAAGGAC (reverse)qRT-PCR for *Cxcr5*
AGGTCGGTGTGAACGGATTTG (forward)GGGGTCGTTGATGGCAACA (reverse)qRT-PCR for *Gapdh*

### Mouse Genotyping

A mix of three primers was used to genotype Rosa26 reporter mice as previously described [22]. The genotyping of *TgUICBAC* mice was done by PCR using primers that amplified a segment of *iCreERT2*, and by Southern blot using a probe outside of the *Upk2* gene to identify both the endogenous allele and the transgene. PCR was performed at the following conditions using HotStarTaq Master Mix (Qiagen, Valencia, CA): 95°C for 15′, 30 cycles of 95°C for 30″, 57°C for 1′, 72°C for 1′ and 72°C for 5′ for the last cycle yielding a 312-bp product for the transgenic allele. Southern Blot was performed following standard procedures. Briefly, mouse tail genomic DNA was extracted using proteinase K digestion and ethanol precipitation. After ScaI digestion, the DNA fragments were resolved by agarose gel electrophoresis, transferred to a hybond membrane (GE Healthcare, Piscataway, NJ), and hybridized with the ^32^P-labeled 389-bp DNA probe located upstream of the mouse *Upk2* promoter. After autoradiography, the copy number of the transgene was determined by comparing the relative density of the transgene with that of the endogenous *Upk2* gene. PCR was used for routine genotyping for the following generations of transgenic mice. Primers and probes used in these procedures are summarized in Table 1.

### RT-PCR and qRT-PCR Analyses

Total RNA was isolated from major organs of *TgUICBAC* mice (both sexes) by QIAzol (Qiagen) according to the manufacturer’s instructions. The RNA integrity was verified by electrophoresis on a 1.2% agarose gel and was visualized with ethidium bromide staining. The concentration was quantified by ultraviolet absorption with a NanoDrop spectrophotometer (Thermo Scientific). One µg of total RNA from each sample was subjected to first-strand cDNA synthesis using SuperScript™III First-Strand Synthesis System for RT-PCR (Invitrogen). RT-PCR was carried out using the GeneAmp PCR System 9700 (Applied Biosystems). Two µl of obtained cDNA were used as template for amplification of a fragment of the iCreERT2. Gapdh was used as a control[26]. PCR was performed at the following conditions using HotStarTaq Master Mix (Qiagen): 95°C for 15′, 30 cycles of 95°C for 30″, 57°C for 1′, 72°C for 1′ and 72°C for 5′ for the last cycle. PCR products were analyzed on 2% agarose gels.

qRT-PCR was conducted using the Mastercycler epgradient S realplex^4^ (Eppendorf) in accordance to the manufacturer’s instructions. RNA transcript levels of *Foxr1, Bcl9l* and *Cxcr5* from 6 *TgUICBAC* bladders (3 females and 3 males) were compared to 6 wild type bladders (3 females and 3 males). qRT-PCR contained in a final volume of 20 µl, 10 µl of 2x QuantiTect SYBR Green PCR Master Mix (Qiagen), 1 µl of cDNA, and 2 µl of the forward and reverse primers. The thermal cycling conditions were: 95°C for 15′, and 40 cycles of 94°C for 30″, 56°C for 40″ and 72°C for 30″. The PCR products were analyzed by melting curve analysis and agarose gel electrophoresis to confirm that no by-products were formed. The relative concentrations of the PCR products derived from the target gene were calculated using the *realplex* software (Eppendorf) using the ΔΔC_T_ method [27]. The results were expressed relative to the levels of a housekeeping gene *Gapdh*. All experiments were conducted in triplicate. Primers used in these procedures are summarized in Table 1.

### LacZ Staining

*TgUICBAC* mice were crossed with Rosa26 reporter mice and the compound mice were subjected to administration of Tamoxifen (Sigma-Aldrich, i.p., 0.15 mg/g body weight, in 15mg/ml of sun flower oil, for 3 consecutive days) or of equal volume of sun flower oil (control group). One week after the last injection, mice were euthanized, major organs were taken out and embedded in OCT. X-gal staining was performed following standard protocol. Briefly, frozen 10 µm sections were fixed with 4% paraformaldehyde in PBS (pH = 7.4) for 10 minutes at 4°C. Then sections were stained with 1mg/ml X-gal in PBS (pH = 7.4) containing 5mM K_4_Fe(CN)_6_, 5mM K_3_Fe(CN)_6_, 2mM MgCl_2,_ 0.1% NP-40, and 0.1% sodium deoxycholate at 37°C for 15 hours. After being washed in PBS, sections were fixed with 10% formalin for 10 minutes, counterstained with nuclear fast red for 5 minutes and mounted with an aqueous mounting medium.

### Immunofluorescence Analysis and Recombination Quantification

Double immunofluorescence analysis was performed on 10-µm frozen tissue sections from four Tamoxifen injected mouse bladder samples (2 females and 2 males) to determine the rate of co-expression of Upk2 and ß-galactosidase. Sections were fixed for 10 minutes with 4% Paraformaldehyde and after washing in 1X PBS submitted to antigen retrieval by steam treatment for 15 minutes in 10 mM citrate buffer, pH 6.0. Slides were then treated with 10% normal horse serum for 30 minutes, followed by ß-galactosidase antibody (mouse monoclonal 40–1a, Santa Cruz) incubation overnight at 4°C. Slides were then incubated with biotinylated secondary antibodies (Vector Laboratories) for 30 minutes, followed by strepatvidin-594 (Invitrogen) incubation for 60 minutes. After that, slides were blocked with 10% normal donkey serum for 30 minutes, followed by Upk2 antibody (goat polyclonal, Santa Cruz) incubation overnight at 4°C. Slides were then incubated with Alexa Fluor® 488 IgG secondary antibodies (Invitrogen) for 60 minutes and then mounted with Vectashileld mounting medium containing DAPI (Vector Laboratories). The antibodies against Upk2 and ß-galactosidase were specific, because urothelial staining was absent when they were omitted as the first antibodies.

For quantification of recombination, we determined the percentage of expression of β-galactosidase in Upk2-expressing cells identified in 5 microscopic high power fields (HPF) of each bladder section. Specifically, in each of the 4 bladder samples analyzed, random areas containing urothelium were identified by DAPI staining at low magnification, and then 5 HPF were analyzed by counting the number of cells displaying a Upk2-positive phenotype and then counting the cells with β-galactosidase positivity. Average of recombination was assessed for the 2 female and 2 male samples separately.

### Statistical Analysis

Statistical analysis was done with IBM SPSS Statistics v19.0. t-test was used to assess differences in transcript expression of *Foxr1, Bcl9l* and *Cxcr5* genes between bladders of wild type and *TgUICBAC* mice, and to assess differences of co-expression of Upk2 and β-galactosidase in female and male bladder urothelium. All statistical tests were 2-sided with significance considered at p = 0.05.

## Supporting Information

Figure S1 **qRT-PCR analysis of relative expression levels of** ***Bcl9l, Cxcr5*** **, and** ***Foxr1*** **in the bladders of Wt (set to 1) and** ***TgUICBAC*** **mice.** Mouse *Gapdh* expression level was used as control. Refer to “Method” for more detail. In a brief summary, *Bcl9l* showed a 2.2-fold increase in male (p = 0.014) and 1.8-fold increase in female (p = 0.02); *Cxcr5* showed a 20.8-fold increase in male (p = 0.013) and 21.7-fold increase in female (p = 0.002); and *Foxr1* showed a 40.0-fold increase in male (p = 0.033) and 43.0-fold increase in female (p = 0.006). No significant differences were observed between male and female *TgUICBAC* mice in transcript levels of the three genes.
(TIF)
