# Supplementary figures and images for: A BAC-Based Transgenic Mouse Specifically Expresses an Inducible Cre in the Urothelium
Source: PLoS One. 2012 Apr 9;7(4):e35243. doi: 10.1371/journal.pone.0035243 (PMC3322165; doi:10.1371/journal.pone.0035243)

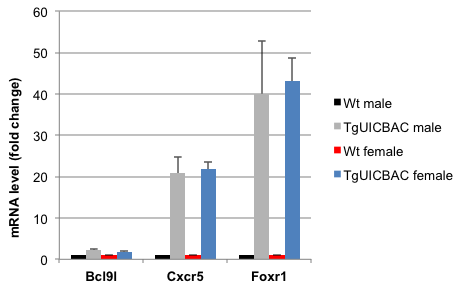

Supplement: Figure S1 — qRT-PCR analysis of relative expression levels of Bcl9l, Cxcr5 , and Foxr1 in the bladders of Wt (set to 1) and TgUICBAC mice. Mouse Gapdh expression level was used as control. Refer to “Method” for more detail. In a brief summary, Bcl9l showed a 2.2-fold increase in male (p = 0.014) and 1.8-fold increase in female (p = 0.02); Cxcr5 showed a 20.8-fold increase in male (p = 0.013) and 21.7-fold increase in female (p = 0.002); and Foxr1 showed a 40.0-fold increase in male (p = 0.033) and 43.0-fold increase in female (p = 0.006). No significant differences were observed between male and female TgUICBAC mice in transcript levels of the three genes. (TIF) [file pone.0035243.s001.tif]
